# People's self-reported encounters of Perceiving Mind in Artificial Intelligence

**DOI:** 10.1016/j.dib.2019.104220

**Published:** 2019-07-06

**Authors:** Daniel B. Shank, Alexander Gott

**Affiliations:** Department of Psychological Science, Missouri University of Science and Technology, 500 W. 14th Street, Rolla, MO 65409, USA

**Keywords:** Emotions, Artificial intelligence, Mind, Mind perception, Algorithms

## Abstract

This article presents the data from two surveys that asked about everyday encounters with artificial intelligence (AI) systems that are perceived to have attributes of mind. In response to specific attribute prompts about an AI, the participants qualitatively described a personally-known encounter with an AI. In survey 1 the prompts asked about an AI planning, having memory, controlling resources, or doing something surprising. In survey 2 the prompts asked about an AI experiencing emotion, expressing desires or beliefs, having human-like physical features, or being mistaken for a human. The original responses were culled based on the ratings of multiple coders to eliminate responses that did not adhere to the prompts. This article includes the qualitative responses, coded categories of those qualitative responses, quantitative measures of mind perception and demographics. For interpretation of this data related to people's emotions, see *Feeling our Way to Machine Minds: People's Emotions when Perceiving Mind in Artificial Intelligence* Shank et al., 2019.

**Table:** Specifications table

Subject area	*Psychology*
More specific subject area	*Social Psychology; Psychology of Technology; Mind Attributions*
Type of data	*Excel file, word codebook, tables*
How data was acquired	*Online Qualtrics surveys in 2018 to US residents using Amazon Mechanical Turk*
Data format	*Raw, analyzed*
Experimental factors	*Recruited participants on Amazon Mechanical Turk by advertising the nature of the surveys*
Experimental features	*Online participants selected a prompt to answer, answered it in an essay box, listed the names of the primary interactants, then answered morality and mind measures, demographics, and a moral foundations theory questionnaire.*
Data source location	*United States*
Data accessibility	*Data is within this article and the supplemental material*
Related research article	Shank, D. B., Graves, C., Gott, A., Gamez, P., & Rodriguez, S. (2019). Feeling Our Way to Machine Minds: People's Emotions when Perceiving Mind in Artificial Intelligence. *Computers in Human Behavior, 98, 256–266.*https://doi.org/10.1016/j.chb.2019.04.001*.*

**Table:** 

**Value of the data** • This data can be used as a historical reference point for the types of encounters with AIs where people perceive the AI as minded in 2018 • The qualitative responses can be analyzed with qualitative software or techniques to uncover patterns and commonalities in encounters with minded AIs • The quantitative measures can be used to compare or used in a meta-analysis of perceived mind measures • The qualitative responses can be adapted for use as stimuli of real-world encounters with AIs

## 1 Data

The data is from two surveys which asked participants to qualitatively describe a personally-known situation with an artificial intelligence (AI) in response to a prompt that asked about an aspect related to the AI having mind. The participants were allowed to select which prompt to answer from four options (Table 1). The participants answered additional follow-up questions about who/what was involved in the scenario, demographics, perceived mind (Table 2), and further completed a moral foundations theory questionnaire. The qualitative responses were then coded into categories in terms of AI type, affect expressed by the human, prompt adherence, and task (or situation) types (refer to codebook material for survey details and dataset for participant and coding data).

**Table 1:** Prompt wording and response count.

Code	Survey	Prompt	Responses
1	1	Describe a personal interaction with an Artificial Agent where it seemed to make sophisticated decisions or plan.	20
2	1	Describe a personal interaction with an Artificial Agent where it appeared to act on its own memory of the past.	30
3	1	Describe a personal interaction with an Artificial Agent where it was in control of resources, information, or an outcome.	46
4	1	Describe a personal interaction with an Artificial Agent where it did something unexpected or surprising.	63
5	2	Describe a personal interaction with an Artificial Agent where it seemed to experience pain, pleasure, or distress.	14
6	2	Describe a personal interaction with an Artificial Agent where it seemed to express its own desires or beliefs.	21
7	2	Describe a personal interaction with an Artificial Agent where it had human-like physical features.	22
8	2	Describe a personal interaction with an Artificial Agent where it was mistaken for a human.	50

**Table 2:** Means and standard deviation of sixteen items to measure mind perception and two items to measure surprise.

Measure	Range	Survey 1 Mean (SD)	Survey 2 Mean (SD)
[AI] has a mind of its own	1 to 5	2.26 (1.40)	2.53 (1.36)
[AI] has intentions	1 to 5	2.48 (1.40)	2.97 (1.39)
[AI] can plan actions	1 to 5	3.29 (1.37)	3.42 (1.26)
[AI] can recognize emotion	1 to 5	1.90 (1.13)	2.68 (1.36)
[AI] can act in order to meet its goals	1 to 5	3.21 (1.42)	3.62 (1.19)
[AI] can remember the past	1 to 5	3.18 (1.42)	3.11 (1.40)
[AI] can reason	1 to 5	2.65 (1.33)	3.04 (1.37)
[AI] has desires	1 to 5	1.68 (1.13)	2.29 (1.32)
[AI] has beliefs	1 to 5	1.70 (1.12)	2.30 (1.29)
[AI] can have experiences	1 to 5	2.02 (1.24)	2.55 (1.36)
[AI] can experience emotional pain or pleasure	1 to 5	1.48 (0.89)	1.92 (1.17)
[AI] has a personality	1 to 5	2.15 (1.30)	3.25 (1.32)
[AI] can feel anticipation	1 to 5	1.92 (1.29)	2.33 (1.34)
[AI] seeks continued functioning	1 to 5	2.94 (1.49)	3.29 (1.35)
[AI] can feel distress	1 to 5	1.55 (0.92)	2.03 (1.20)
[AI] can recognize sensations	1 to 5	1.78 (1.16)	2.24 (1.31)
How surprising were the behaviors of [AI] in the event you described?	1 to 3	2.09 (0.71)	2.18 (0.66)
How much did the event you described initially surprise you?	1 to 3	2.17 (0.71)	2.33 (0.63)

## 2 Experimental design, materials, and methods

### 2.1 Participants and design

Survey 1 was conducted on 4/13/2018 and survey 2 on 7/16/2018. Both surveys were conducted through Amazon Mechanical Turk, a crowd sourcing website often used in social science research [7], [9]. To participate, Amazon Mechanical Turk workers had to have a positive history (100 HITs, 90% approval rating) and be located in the United States. Survey 1 included 183 initial responses and survey 2 included 127 initial responses (initial responses only include ones where the prompt was actually coherently answered). A culling process, detailed below, was used to eliminate responses that did not conform to the instructions and prompts. This reduced the survey 1 data to 159 responses and survey 2 data to 107 responses. Participants were 70.7% white with a mean age of 34.7, and included 135 men, 129 women, and 2 who did not report gender. The supplementary data includes the complete culled dataset and codebook.

### 2.2 Prompts and prompt responses

Instead of asking about artificial intelligence, participants were shown the following definition of “artificial agents” twice – once when selecting the prompt and once when answering the prompt:

An Artificial Agent is any computer, computer program, device, app, machine, robot, bot, or sim that performs behaviors which are considered intelligent if performed by humans, learns or changes based on new information or environments, generalizes to make decisions based on limited information, or makes connections between otherwise disconnected people, information, or other agents.

In the same two places they were also shown a definition of personal interaction: “A personal interaction is any interaction that you, a family member, or friend had or that you personally witnessed.”

Participants were allowed to select one from a choice of four randomly-ordered prompts for each survey (Table 1). Prompts in survey 1 were designed to relate to components of agentic mind, whereas prompts in survey 2 were designed to relate to components of experiential mind. Agentic mind is the capacity to act, have intention, and plan, whereas experiential mind is the capacity to feel emotions or pain, desires, and experiences [4], [6], [13]. The first two prompts in each survey (i.e., prompts 1 and 2, 5 and 6) were directly based on the standard measures of agentic and experiential minds. The last two prompts in each survey (i.e., prompts 3 and 4, 7 and 9) were related to situations that produce increased perceptions of agentic and experiential mind, respectively, but are not part of their standard measures. The prompt wording and number of responses are displayed in Table 1.

Participants responded to the prompt with an essay box and two further qualitative questions about the response: “What is the name that you would call the Artificial Agent in this event?” and “Who or what did the Artificial Agent primarily interact with in this event?” The answers to these were primarily used to populate the quantitative questions and defaulted to “The Artificial Agent” and “The Other Interactant”, respectively.

### 2.3 Follow-up quantitative measures

After the prompt responses, participants first responded to questions about moral violations in the incident according to each foundation in moral foundations theory [3]. Next, participants answered a question on the source of the moral violation if any. Next, participants completed 16 items measuring mind perception toward the AI – 7 items related to agentic mind and 9 items related to experiential mind (Table 2). These mind measures were adapted from other studies [1], [2], [5], [8], [10], [12]. Following this were two questions on surprise (Table 2).

The next and final page first included measures of gender, race, age, education. Following those were moral foundation theory personality items. Finally, there were two questions about technology knowledge and interaction. The question wording and answer options are in the codebook and the responses in the dataset, both in the supplementary material.

### 2.4 Culling

Three student researchers were coders of the qualitative data; one was an author on the research paper and the others were simply hired as coders. They were instructed on the coding procedure, conducted test cases, and met with the research team to confirm that all coders were proceeding correctly and interpreting the coding similarly. The coders then did their coding independently of each other in Microsoft Excel without any feedback during the coding process. The coders used three criteria to evaluate each of the responses and then based on those criteria made an overall recommendation of exclude, include, or unsure. The criteria involved the degree to which the response (1) contained an AI (or artificial agent as defined previously), (2) involved a personal interaction (as defined previously), and (3) responded to the set of prompts for each survey. Each coder was instructed to rate each of the three criteria as low (0 or 1), medium (2 or 3) or high (4 or 5). An example of a medium score on the first criteria would be when the response contained digital technology, but clearly not an AI. An example of a medium score on the second criteria would be an interaction that the respondent did not witness, but affected the respondent second-hand. An example of a medium score on the third criteria would be when there was not enough information in the response to confirm it was clearly addressing the prompt, but nothing that indicated it was not.

The coders were instructed that any response with high scores across all three criteria should receive an include recommendation, whereas any response with even one low score should receive an exclude recommendation. For other combinations of scores the coders used their discretion based on their overall evaluation. For 83.9% of the responses there was agreement (three recommendations agreeing on exclude/include, or two recommendations agreeing on exclude/include with one unsure). The remaining 16.1% of the cases were coded by two additional authors (one student and one professor) which nearly always led to a recommendation based on majority vote among the five coders. In the few cases with ties, the final two coders decided through discussion.

### 2.5 Coding for categories

The qualitative responses were also coded into categories to allow for additionally analyses. Each category was determined through examination of the data and discussion and each was coded by one of the same four student coders involved in the culling. The first coder listed all emotions and affective expressions from the qualitative response that were expressed by the interacting person (see [11] for details). Note that sometimes the exact word listed was not actually present in the original response, but was implied. The second coder cycled through the responses and identified 76 types of AIs in the responses. Three other coders coded the responses in terms of these types, however their agreement was less than 50% due to many of the 76 types overlapping in features and attributes (e.g., a game AI on a device). Therefore, the second coder reduced the categories from 76 down to a final classification of eight overarching AI type categories – customer service, bots, game AIs, robots and machines, smart assistants, smart devices, software, and other. The second coder placed each response's AI in one of these categories based on the previous coding into the 76 categories.

The third coder made non-exclusive binary classifications of the task or situation type reported in the response. These classifications include if the person was (1) testing or messing around with the AI, (2) interrupted by the AI, (3) accessing entertainment, (4) performing a personal task, (5) engaging in a routine, (6) performing a business transaction, and if the AI (7) succeeded in a task and (8) failed at a task. The fourth coder, blind to the prompt being responded to, coded each response in terms of its fitting each of the eight prompts. This is similar to a manipulation check, but also allows one to see which mind perception attributes were related to the prompt questions not chosen (see [11] Table 1).
