# Supplementary material for: People's self-reported encounters of Perceiving Mind in Artificial Intelligence
Source: Data Brief. 2019 Jul 6;25:104220. doi: 10.1016/j.dib.2019.104220 (PMC6646919; doi:10.1016/j.dib.2019.104220)
Supplement: Supplementary file 2 [file mmc2.docx]

Shank et al. Codebook

This codebook goes through the surveys in sequential order excluding the consent form and Amazon Mechanical Turk instructions. It reproduces all information/instructions, question prompts, question answer choices, and coding of those choices in parentheses.

**Opening Page.**

An Artificial Agent is any computer, computer program, device, app, machine, robot, bot, or sim that performs behaviors which are considered intelligent if performed by humans, learns or changes based on new information or environments, generalizes to make decisions based on limited information, or makes connections between otherwise disconnected people, information, or other agents.

A personal interaction is any interaction that you, a family member, or friend had or that you personally witnessed.

**Question (Survey 1 only; choices displayed in random order).** Which prompt would you like to respond to?

- Describe a personal interaction with an Artificial Agent where it seemed to make sophisticated decisions or plan. (1)
- Describe a personal interaction with an Artificial Agent where it appeared to act on its own memory of the past. (2)
- Describe a personal interaction with an Artificial Agent where it was in control of resources, information, or an outcome. (3)
- Describe a personal interaction with an Artificial Agent where it did something unexpected or surprising. (4)

**Question (Survey 2 only; choices displayed in random order).** Which prompt would you like to respond to?

- Describe a personal interaction with an Artificial Agent where it seemed to experience pain, pleasure, or distress. (5)
- Describe a personal interaction with an Artificial Agent where it seemed to express its own desires or beliefs. (6)
- Describe a personal interaction with an Artificial Agent where it had human-like physical features. (7)
- Describe a personal interaction with an Artificial Agent where it was mistaken for a human. (8)

**Qualitative Response Page.**

An Artificial Agent is any computer, computer program, device, app, machine, robot, bot, or sim that performs behaviors which are considered intelligent if performed by humans, learns or changes based on new information or environments, generalizes to make decisions based on limited information, or makes connections between otherwise disconnected people, information, or other agents.

A personal interaction is any interaction that you, a family member, or friend had or that you personally witnessed.

Try to write at least 4-6 sentences about the details of the interaction event, including what happened before and after it, who and what was involved, who witnessed it, and the outcome based on the following prompt:

***[Prompt Inserted Here]***

***Qualitative Response:***

**Question (Default text present: “The Artificial Agent”).** What is the name that you would call the Artificial Agent in this event?

________________________________________________________________

**Question (Default text present: “The Other Interactant”)** Who or what did the Artificial Agent primarily interact with in this event?

________________________________________________________________

**Additional Questions Page.**

Please answer a few questions about the interaction you reported.

**Questions (randomized order).** For this event, how much did each of the following occur?

|  | Not at all (1) | (2) | Somewhat (3) | (4) | Very much (5) |
| --- | --- | --- | --- | --- | --- |
| Harm or causing pain |  |  |  |  |  |
| Unfairness or cheating |  |  |  |  |  |
| Disloyalty or betrayal |  |  |  |  |  |
| Disrespecting or disobeying authority |  |  |  |  |  |
| Degrading or impure actions |  |  |  |  |  |
| Restriction of liberty or loss of privacy |  |  |  |  |  |
| A moral violation or wrong |  |  |  |  |  |

**Question.** If any moral violation or wrong occurred, who or what appears to be the primary cause of it?

- ***[The Artificial Agent inserted here]*** (1)
- ***[The Other Interactant inserted here]*** (2)
- Both ***[The Artificial Agent inserted here]*** and ***[The Other Interactant inserted here]*** (3)
- Someone or something else (4)
- No one or not applicable (5)

**Question (randomized order).** How much do you agree with each of the following?

|  | Completely disagree (1) | (2) | Neither agree nor disagree (3) | (4) | Completely agree (5) |
| --- | --- | --- | --- | --- | --- |
| ***[The Artificial Agent inserted here]*** has a mind of its own (1) |  |  |  |  |  |
| ***[The Artificial Agent inserted here]*** has intentions (2) |  |  |  |  |  |
| ***[The Artificial Agent inserted here]*** can plan actions (3) |  |  |  |  |  |
| ***[The Artificial Agent inserted here]*** can recognize emotions (4) |  |  |  |  |  |
| ***[The Artificial Agent inserted here]*** can act in order to meet its goals (5) |  |  |  |  |  |
| ***[The Artificial Agent inserted here]*** can remember the past (6) |  |  |  |  |  |
| ***[The Artificial Agent inserted here]*** can reason (7) |  |  |  |  |  |
| ***[The Artificial Agent inserted here]*** has desires (8) |  |  |  |  |  |
| ***[The Artificial Agent inserted here]*** has beliefs (9) |  |  |  |  |  |
| ***[The Artificial Agent inserted here]*** can have experiences (10) |  |  |  |  |  |
| ***[The Artificial Agent inserted here]*** can experience emotional pain or pleasure (11) |  |  |  |  |  |
| ***[The Artificial Agent inserted here]*** has a personality (12) |  |  |  |  |  |
| ***[The Artificial Agent inserted here]*** can feel anticipation (13) |  |  |  |  |  |
| ***[The Artificial Agent inserted here]*** seeks continued functioning (14) |  |  |  |  |  |
| ***[The Artificial Agent inserted here]*** can feel distress (15) |  |  |  |  |  |
| ***[The Artificial Agent inserted here]*** can recognize sensations (16) |  |  |  |  |  |

**Question.** How surprising were the behaviors of ***[The Artificial Agent inserted here]*** in the event you described?

- Not surprising (1)
- Somewhat surprising (2)
- Extremely surprising (3)

**Question.** How much did the event you described initially surprise you?

- I was not at all surprised (1)
- I was somewhat surprised (2)
- I was extremely surprised (3)

**Final Question Page.**

You have finished answering questions about the interaction.  Please answer a few final questions.

**Question.** What is your gender?

- Male (1)
- Female (2)

**Question.** What is your race?

- African American/Black (1)
- Asian/Pacific Islander (2)
- Caucasian/White (3)
- Hispanic (4)
- Native American/Alaska Native (5)
- Other/Multi-Racial (6)
- Decline to respond (7)

**Question.** What is your age?

________________________________________________________________

**Question.** What is your highest level of education?

- Some high school (1)
- Graduated high school (2)
- Some college (3)
- Associate's degree (4)
- Bachelor's degree (5)
- Some graduate school (6)
- Master's degree (7)
- Ph.D./Terminal Professional degree (8)

**Question.** When you decide whether something is wrong, to what extent are the following considerations relevant to your thinking.

|  | Not at all relevant (1) | (2) | Neutral (3) | (4) | Extremely relevant (5) |
| --- | --- | --- | --- | --- | --- |
| Whether someone suffered emotionally |  |  |  |  |  |
| Whether some people were treated differently than others |  |  |  |  |  |
| Whether someone's actions showed love for his or her country |  |  |  |  |  |
| Whether someone disrespected an authority |  |  |  |  |  |
| Whether someone violated standards of purity and decency |  |  |  |  |  |
| Whether someone restricted another's freedom |  |  |  |  |  |
| Whether someone cared for someone weak or vulnerable |  |  |  |  |  |
| Whether someone acted unfairly |  |  |  |  |  |
| Whether someone did something to betray his or her group |  |  |  |  |  |
| Whether someone conformed to the traditions of society |  |  |  |  |  |
| Whether someone did something disgusting |  |  |  |  |  |
| Whether someone made public something that another wished to keep private |  |  |  |  |  |

**Question.** How familiar are you with personally interacting with Artificial Agents?

- Extremely familiar (1)
- Very familiar (2)
- Moderately familiar (3)
- Slightly familiar (4)
- Not familiar at all (5)

**Question.** How familiar are you with developing software, writing code, or building robots?

- Extremely familiar (1)
- Very familiar (2)
- Moderately familiar (3)
- Slightly familiar (4)
- Not familiar at all (5)
